# The predictive value of molecular markers (p53, EGFR, ATM, CHK2) in multimodally treated squamous cell carcinoma of the oesophagus

**DOI:** 10.1038/sj.bjc.6604037

**Published:** 2007-10-16

**Authors:** M Sarbia, N Ott, F Pühringer-Oppermann, B L D M Brücher

**Affiliations:** 1 Department of Pathology, Unfallkrankenhaus Berlin, Berlin, Germany; 2 Department of Pathology, Technical University of Munich, Munich, Germany; 3 Department of Surgery, Technical University of Munich, Munich, Germany

**Keywords:** squamous cell carcinoma, oesophagus, immunohistochemistry

## Abstract

Pretherapeutic identification of oesophageal squamous cell carcinomas that will respond to neoadjuvant chemoradiotherapy is an important attempt for improvement of patient's prognosis. In the current study, pretherapeutic biopsies from 94 oesophageal squamous cell carcinomas (cT3, cN0/+, cM0) in patients who underwent neoadjuvant chemoradiotherapy (RCTx: 45 Gy plus cisplatin and 5-fluorouracil) and subsequent oesophagectomy in the setting of a single-centre prospective treatment trial were investigated by means of immunohistochemistry. Expression of proteins involved in DNA repair and/or cell-cycle regulation, that is p53, p53 (phosphorylated at Ser15), EGFR, ATM protein kinase (phosphorylated at Ser1981) and checkpoint kinase 2 (CHK2) (phosphorylated at Thr68) was correlated with the response to RCTx and with overall survival. Tumours that were positive for CHK2 expression more frequently showed clinically determined regression after RCTx (69.4%) than tumours that were negative for CHK2 expression (32.1%; *P*=0.0011), whereas other parameters did not correlate with tumour regression. Expression of ATM correlated with expression of CHK2 (*P*=0.0061) and p53-phospho (*P*=0.0064). Expression of p53 correlated with expression of p53-phospho (*P*<0.0001). In contrast to clinical and histopathological response evaluation, none of the molecular parameters under investigation correlated with overall survival. In conclusion, expression analysis of p53, EGFR CHK2 and ATM has no predictive value in multimodally treated oesophageal squamous cell carcinoma.

The prognosis for patients with oesophageal squamous cell carcinoma has only improved slightly in recent years. The results of surgical therapy have been poor, with 5-year survival rates varying between 9 and 40%, even with lesions in resectable stages (Enzinger and Mayer, 2003). Combined treatment modalities – including chemotherapy, radiotherapy and surgical treatment – have therefore been investigated in increasing number of studies to improve the survival of patients with oesophageal cancer. These studies have indicated a complete response in 20–40% of patients preoperatively treated with combined radiotherapy and chemotherapy (Ilson, 2004). However, with regard to survival, the benefit of combined neoadjuvant treatment modalities has only been confirmed unequivocally in the subset of patients who have a complete response at histopathological examination (Lerut *et al*, 1999; Ancona *et al*, 2001; Brücher *et al*, 2004). Finding parameters that might help identify those patients capable of benefiting from multimodal treatment modalities before the start of therapy would therefore be of considerable interest (Lerut *et al*, 1999; Ilson, 2004).

In the present study, an immunohistochemical assessment was therefore carried out of the expression of a panel of genes (*p53*, *EGFR*, *ATM* and *CHK2*) that are potentially involved in the response to chemoradiotherapy. Expression analysis was based on pretherapeutic tumour biopsies from 94 patients who had locally advanced oesophageal squamous cell carcinoma and received multimodal treatment. Subsequently, protein expression was correlated with the response to chemoradiotherapy and with overall survival.

## MATERIALS AND METHODS

### Patients

All of the patients included in the present investigation were participants in prospective single-centre phase II studies conducted at the Rechts der Isar Hospital, Technical University of Munich, Germany. The results of these studies, together with the criteria for patient selection and study design, have been previously described elsewhere (Brücher *et al*, 2004). Patients who had previously undergone chemotherapy, radiotherapy, laser therapy or stent implantation were excluded from this study. Informed consent was obtained from all patients.

Patients with histologically proven, locally advanced intra-thoracic (*n*=81) or cervical (*n*=13) oesophageal squamous cell cancer, without distant metastases (uT3, uN0/+, cM0), who were considered medically fit for surgery were treated with neoadjuvant chemoradiotherapy (Brücher *et al*, 2004) in consecutive phase II trials between 1999 and 2004, followed by oesophagectomy. The staging procedures in all patients consisted of endoscopy, endoluminal ultrasonography (EUS), bronchoscopy (including brush cytology and biopsy) and computed tomography.

### Neoadjuvant chemoradiotherapy and clinical response evaluation

The neoadjuvant regimen consisted of simultaneous radiotherapy/chemotherapy. External-beam radiotherapy was delivered using a two-opposed-field technique with 10 or 15 MV photons (Siemens Mevatron KD-2) at 2 Gy per fraction per day, five fractions per week, up to the year 2000. Between 1999 and 2000, the total dose was 40 Gy (Brücher *et al*, 2004). The clinical target volume, which initially included all of the locoregional lymph-node stations in addition to the tumour, was modified and reduced to the directly juxtaregional nodes (tumour±4 cm craniocaudally), extended by 1.5 cm in all directions for the planning target volume, taking patient movements into account. The total dose was increased thereafter to 45 Gy using single doses of 1.8 Gy. From this time on, three-dimensional treatment planning with three- to five-field conformal techniques was used. Between 1999 and 2000, 5-fluorouracil at 300 mg m^−2^/day was administered simultaneously with radiotherapy as a continuous infusion for 21 days by means of a systemic venous port system. Between 2000 and 2003, cisplatin (CDDP) was administered in addition to 5-fluorouracil five times weekly in weeks 1 and 5, at a dosage of 20 mg m^−2^. As of 2003, patients were enrolled in a phase I/II dose-finding study in which oxaliplatin 40–50 mg m^−2^, administered once weekly, replaced CDDP. The dosage of 5-fluorouracil was reduced to 225 mg m^−2^ per day, given as a continuous infusion. No significant impact on clinical outcome was detectable in relation to differences of neoadjuvant therapy protocols (data not shown).

All of the patients underwent clinical response evaluation as described in detail earlier. Briefly, clinical response evaluation was carried out 3–4 weeks after completion of multimodal therapy, using repeated endoscopy and computed tomography in accordance with the World Health Organization criteria (Miller *et al*, 1981). In detail, response as assessed by endoscopy was determined by one investigator. Categorisation was based on macroscopic aspects of the intra-luminal tumour mass. Endoluminal ultrasonography had not been used for clinical response evaluation, since own experiences have shown lack of diagnostic accuracy after radiochemotherapy (Brücher *et al.* 2004). Additionally, response assessment according to the computed tomography scan was performed by one experienced radiologist, measuring maximal tumour length and maximum wall thickness before and after treatment. Clinical findings as well as the results of endoscopy and radiology were discussed by the investigators and response was defined in consensus according to the WHO criteria: clinical complete response, clinical partial response, clinical minimal response and clinical no change. For statistical analysis, the clinical response was divided into two groups: group 1 (clinical responder) consisted of patients with clinical complete response and clinical partial response, while group 2 (clinical nonresponder) included patients with clinical minimal response and clinical no change.

### Surgical therapy

Surgery was performed on all patients within 1 week after the clinical response evaluation. The patients underwent standardised transthoracic *en bloc* oesophagectomy with two-field lymphadenectomy (Siewert *et al*, 2001). Gastrointestinal continuity was restored with a two-stage gastric pull-up procedure, as described previously (Brücher *et al*, 2006). To avoid a high rate of paresis of the recurrent nerves, lymph-node dissection in the neck was not performed. Cervical tumours were treated with a partial oesophageal resection and reconstructed with a free jejunal graft (Brücher *et al*, 2007).

### Histopathological response evaluation

The resection specimens were fixed in formaldehyde (4%) for 24 h. The complete tumour was embedded in paraffin blocks. Sections of each paraffin block were cut (5 *μ*m) and stained with haematoxylin and eosin. The tumour stage was classified in accordance with the UICC criteria (Wittekind *et al*, 2002). Accordingly, 34 tumours were categorised ypT0, 7 ypT1, 23 ypT2, 28 ypT3 and 2 ypT4. Regarding nodal status, 62 tumours were in category ypN0 and 32 in ypN1. The histopathological response to chemoradiotherapy was classified in accordance with criteria published previously (Becker *et al*, 2003; Brücher *et al*, 2006). The percentage of viable residual tumour cells was estimated, and individual patients were subsequently allocated to one of the following groups: no residual tumour cells; <10% residual tumour cells/tumour area; 10–50% residual tumour cells; and >50% residual tumour cells.

### Pathological review of pretherapeutic tumour biopsies

The tumour biopsies obtained endoscopically during the pretherapeutic staging procedures were retrieved from the files of the Institute of Pathology at the Technical University of Munich, with approval from the local ethics committee. Of the 94 patients, 71 were male and 23 were female. Their median age was 59 years (range, 19–76). Histological slides of the biopsy specimens were stained with haematoxylin and eosin to determine the tumour type and tumour grade (WHO) by one of the authors (MS). Thirty-two tumours were graded as G2 and 62 tumours were graded as G3.

### Immunohistochemical investigations based on pretherapeutic tumour biopsies

Fresh 4-*μ*m sections from the pretherapeutic biopsies were transferred to glass slides, dewaxed and rehydrated. Antigen retrieval methods (e.g., microwave oven heating in citrate-buffered saline) were used in accordance with the manufacturers' recommendations. Subsequently, the slides were incubated with the primary antibodies. Detailed data on the antibodies are given in Table 1. For three of the four proteins being analysed – that is, p53, ATM, and CHK2 – antibodies were used that detect phosphorylation at specific amino-acid residues that indicate functional activity (ATM, CHK2) (Bakkenist and Kastan 2003; Bartkova *et al*, 2005) or resistance against inactivation (p53) (Shieh *et al*, 1997). The reaction was developed using the labelled streptavidin–biotin–alkaline phosphatase system, with fast red as chromogen. After counterstaining with haematoxylin, the slides were dehydrated in increasing concentrations of ethanol and mounted. For positive controls, tissues with known expression of the respective antigens were used (p53 and EGFR: oesophageal squamous cell carcinoma; ATM and CHK2: colorectal carcinoma). For negative controls, irrelevant antibodies with the same immunoglobulin isotype were used.

The slides were reviewed by a single pathologist (MS) who was blinded to the results of chemoradiotherapy and the patients' survival data. In an initial approach, the percentage of immunoreactive tumour cells was graded on a scale of 0–4 (0=0–4% positive tumour cells; 1=5–24%; 2=25–49%; 3=50–74%; 4=75–100%). Subsequent correlation analyses – including molecular markers, tumour regression following chemoradiotherapy and overall survival – revealed small groups of patients who were unsuitable for meaningful statistical analysis. None of the statistical analyses showed significant correlations (data not shown). All of the molecular markers were therefore subsequently analysed as binary variables (negative *vs* positive).

### Follow-up

After resection, the patients were examined every 3 months for 2 years at the outpatient clinic, and thereafter at 6-monthly intervals. Follow-up included a physical examination, laboratory investigations, plain chest radiography, upper gastrointestinal endoscopy, abdominal ultrasound and computed tomography of the chest and abdomen. Immobilised patients with low performance status were examined for progress and/or recurrence by their general practitioner. The follow-up was complete, with a median of 5.0±1.5 years (95% CI, 2.3–7.3). Thirty-six of the 94 patients (38.3%) died during the follow-up period.

### Statistical analysis

The SAS software package (SAS Institute Inc., Cary, NC, USA) was used for statistical analysis. Statistical analysis of the correlation between therapy-induced tumour regression and molecular markers (p53, EGFR, ATM, CHK2) was carried out using the two-tailed Fisher's exact test. The parameters were dichotomised as follows: tumour response, <10% residual tumour cells *vs* ⩾10% residual tumour cells; molecular markers were dichotomised as negative *vs* positive. The Kaplan–Meier method for analysing censored data was used to calculate survival rates and tested with the log–rank test. Probabilities <0.05 were considered statistically significant.

## RESULTS

### Clinical and histopathological response after neodjuvant radiochemotherapy

Following completion of neoadjuvant radiochemotherapy, according to clinical response evaluation, 53 cases showed partial response, 40 showed minimal response and 1 showed no change. According to histopathological response evaluation, 34 cases showed no residual tumour cells (complete response), 26 showed <10% residual tumour cells (major response), 23 showed 10–50% residual tumour cells (partial response) and 11 showed >50% residual tumour cells (minor response/no change).

### Immunohistochemical analyses of pretherapeutic tumour biopsies

Nuclear accumulation of p53 protein was found in 77.8% of the cases, and nuclear accumulation of p53 protein, phosphorylated at Ser15, was detectable in 73.3% of the cases. Concordance (either both positive or both negative) between p53 and p53 (phospho-Ser15) was found in 69 cases (82.1%), whereas discordance was present only in 15 cases (17.9%; *P*<0.0001). In addition, expression of p53-phospho correlated with expression of ATM (concordance: 76.3%; *P*=0.0064). Neither the expression of p53 nor of p53 (phospho-Ser15) correlated with any of the other markers under analysis (data not shown).

Nuclear expression of ATM was found in 91.6% of the tumours, whereas nuclear expression of CHK2 was detected in 66.7% of cases. Concordance between ATM and CHK2 was found in 55 cases (72.4%), whereas discordance was present only in 21 cases (27.6%; *P*=0.0061). Expression of neither ATM nor CHK2 correlated with any of the other markers under analysis (data not shown).

Expression of EGFR at the cytoplasmic membrane was detected in 98.9% of tumours, with only one tumour being negative for EGFR expression. No correlation was found between EGFR expression and the expression of any of the other markers.

### Correlation between molecular markers and tumour regression after chemoradiotherapy

Complete or partial tumour regression after chemoradiotherapy, as determined by the clinical follow-up, was observed in 69.6% of the tumours that were positive for CHK2 expression, but only in 32.1% of tumours that were negative for CHK2 expression (*P*=0.011). No correlation was found between clinically determined tumour regression and the expression of the other parameters under investigation (Table 2). In addition, no correlation was detected between histopathologically determined tumour regression and marker expression (Table 3).

### Survival analyses

No correlation was detectable between survival and the expression of the parameters under analysis (Table 4). In contrast, clinical response evaluation (*P*=0.0254) as well as histopathological response evaluation (*P*<0.0001) was significantly correlated with overall survival.

## DISCUSSION

The current study is based on the outcomes of the clinically and pathologically determined response to chemoradiotherapy and overall survival in patients with locally advanced oesophageal squamous cell carcinoma, who were included in single-centre trials conducted at the Technical University of Munich, Germany, between 1999 and 2004. These major determinants of the results of therapy were correlated with the expression of a panel of proteins mainly involved in cell-cycle regulation and DNA repair.

Two of these molecules, p53 and EGFR, have been tested previously as predictive markers for multimodally treated oesophageal cancer. However, the results have been equivocal. Thus, whereas some studies have indicated that *p53* gene mutation and/or p53 protein accumulation in tumour cells may be significantly associated with resistance to multimodal therapy and/or poor survival (Seitz *et al*, 1995; Ribeiro *et al*, 1998; Sarbia *et al*, 1998; Krasna *et al*, 1999; Gibson *et al*, 2003), others have not found such a correlation (Soontrapornchai *et al*, 1999; Ito *et al*, 2001; Shimada *et al*, 2002; Pühringer-Oppermann *et al*, 2006). With regard to EGFR, two studies indicated that tumours with strong EGFR expression show a relatively poor response to chemoradiotherapy (Hickey *et al*, 1994; Gibson *et al*, 2003), whereas another study did not find such a correlation (Miyazono *et al*, 2004). Moreover, in one of the two positive studies (Gibson *et al*, 2003), EGFR was found to have predictive value in oesophageal adenocarcinoma, but not in squamous cell carcinoma.

There may be many reasons for such discrepancies, but two factors appear to be of particular importance – small sample sizes and inhomogeneous patient selection. With regard to the sample size, it should be noted that all but one (Soontrapornchai *et al*, 1999) of the investigations cited above included less than 100 patients in the analysis; the great majority of studies included even less than 50 patients. With regard to patient selection, it is clear that in most studies, patients were treated using a variety of different treatment schedules. In addition, oesophageal adenocarcinomas and squamous cell carcinomas – and even carcinomas of the gastro-oesophageal junction – are often lumped together in clinical trials, although it is considered that important differences with regard to molecular and clinical background as well as to responsiveness to chemoradiotherapy between these tumour types exist.

An attempt was made in the current study to overcome these obstacles, at least partially. For example, a relatively large number of patients (*n*=94) was included, who were selected using homogeneous criteria (including only one tumour type) and treated with a current potentially curative treatment regimen. However, the study clearly failed to show any significant predictive value for the immunohistochemical evaluation of p53 and EGFR. In the case of EGFR, this result is explained by the fact that a nearly 100% positivity rate was found in the biopsy material, so that it was not possible to define prognostic subgroups of a reasonable size. In the case of p53, largely identical results were found when applying the standard antibody (DO-7) for detecting accumulated p53 protein in formalin-fixed tissues and applying a relatively new antibody that detects only p53 that is phosphorylated at Ser15. The latter molecular modification has previously been shown to confer protection to p53 against degradation by Mdm2 and thereby to interfere with the radiosensitivity and/or chemosensitivity of tumour cells (Shieh *et al*, 1997; Gao *et al*, 1999).

Somewhat more promising were the results concerning the other two molecules analysed – that is ATM and CHK2. *ATM,* the gene that is mutated in the hereditary disease ataxia-telangiectasia, codes for a protein kinase that acts as a master regulator of cellular responses to DNA double-strand breaks (Bakkenist and Kastan, 2003). *ATM* is activated in the event of DNA damage – for example, due to exposure to ionising radiation. Several substrates of ATM kinase are involved in radiation-induced cell-cycle arrest at the G_1_ checkpoint, – for example, p53, Mdm2 and CHK2. Moreover, constitutive activation of ATM and CHK2 has recently been demonstrated in a subset of malignant tumours (Bartkova *et al*, 2005). It is therefore tempting to speculate that the activation status of ATM and CHK2 may significantly interfere with the radiosensitivity and/or chemosensitivity of tumour cells (Kastan and Bartek, 2004). However, this hypothesis has not yet been tested clinically in patients with oesophageal cancer. Although a certain degree of correlation was found between the expression of CHK2 and clinically determined tumour regression following chemoradiotherapy, this result largely disappeared when the expression of CHK2 was correlated with the histopathologically determined tumour regression. Since tumour regression can be determined much more exactly by histopathological examination than by clinical methods (Brücher *et al*, 2006), the predictive value of CHK2 expression must be regarded with considerable scepticism. Concerning the potential predictive value of ATM, no impact on tumour regression or overall survival was detectable.

In conclusion, immunohistochemical analysis of the expression of a panel of proteins in pretherapeutic biopsies from a relatively large series of patients with multimodally treated, locally advanced oesophageal squamous cell carcinoma did not reveal unequivocal predictive markers. Additional studies in this field are warranted to allow better identification of those patients who could be benefiting from neoadjuvant therapy for this tumour type.

## Figures and Tables

**Table 1:** Antibodies used in this study

**Marker**	**Clone**	**Company**	**Dilution**	**Buffer, antigen retrieval**
p53	DO-7	Dako, Hamburg, Germany	1 : 200	Citrate, pH 6; heating 7 min
p53, phosphorylated at Ser15	3A36	United States Biological, Swampscott, MA, USA	1 : 75	Citrate, pH 6; heating 7 min
EGFR	31 G7	Cytomed, Baden-Baden, Germany	1 : 60	Protease, 20 min
ATM, phosphorylated at Ser1981	7C10D8	Rockland, Gilbertsville, PA, USA	1 : 1500	Citrate, pH 6; heating 7 min
CHK2, phosphorylated at Thr68	Cat. No. 2661S	Cell Signaling Technology, Danvers, MA, USA	1 : 200	Citrate, pH 6; heating 7 min

**Table 2:** Correlation between the immunohistochemically determined parameters and clinically determined tumour regression following polychemotherapy in patients with multimodally treated oesophageal squamous cell cancer

	**Complete remission/partial remission (%)**	**No change/progression (%)**	***P*-*value***
*p53* a			
Negative	10 (50)	10 (50)	NS
Positive	39 (55.7)	31 (44.3)	
			
*p53* (*phospho*)			
Negative	12 (52.2)	11 (47.8)	NS
Positive	38 (60.0)	25 (40.0)	
			
*EGFR*			
Negative	0 (0)	1 (100)	NS
Positive	49 (56.3)	38 (43.7)	
			
*ATM*			
Negative	4 (57.1)	3 (42.9)	NS
Positive	43 (56.6)	33 (43.4)	
			
*CHK2*			
Negative	9 (32.1)	19 (67.9)	*P*=0.011
Positive	39 (69.6)	17 (30.4)	

NS, no significant correlations according to the *χ*^2^-test.

a For technical reasons (e.g., loss of tissue during immunohistological staining procedures), the number of cases analysable for immunohistochemical analyses may differ from the total number of cases under analysis.

**Table 3:** Correlation between the immunohistochemically determined parameters and tumour regression as determined by histopathological examination following chemoradiotherapy in 94 patients with multimodally treated oesophageal squamous cell cancer^a^

	**<10% residual tumour cells (%)**	**⩾10% residual tumour cells (%)**
*p53*		
Negative^b^	13 (65)	7 (35)
Positive	45 (64.3)	25 (35.7)
		
*p53* (*phospho*)		
Negative	12 (52.2)	11 (47.8)
positive	41 (65.1)	22 (34.9)
		
*EGFR*		
Negative	1 (100)	0 (0)
Positive	55 (63.2)	32 (36.8)
		
*ATM*		
Negative	5 (71.4)	2 (28.6)
Positive	49 (64.5)	27 (35.5)
		
*CHK2*		
Negative	17 (60.7)	11 (39.3)
Positive	39 (69.6)	17 (30.4)

a No significant correlations according to the *χ*^2^-test.

b For technical reasons (e.g., loss of tissue during immunohistological staining procedures), the number of cases analysable for immunohistochemical analyses may differ from the total number of cases under analysis.

**Table 4:** Correlation between immunohistochemically determined parameters and overall survival in patients with multimodally treated oesophageal squamous cell cancer

**Marker, expression**	**Patients (*n*)**	**Censored (*n*)**	**Mean survival (years)**	***P* a**
*p53*				NS
Negative	21	12	2.7	
Positive	70	43	2.6	
				
*p53* (*phospho*)				NS
Negative	23	13	1.9	
Positive	64	39	2.5	
				
*EGFR*				NS
Negative	1	1	—	
Positive	88	52	2.7	
				
*ATM*				NS
Negative	7	4	1.1	
Positive	77	47	2.5	
				
*CHK2*				NS
Negative	28	14	2.7	
Positive	57	38	1.6	

a According to the log–rank test. NS, not significant.

## References

[bib1] Ancona E, Ruol A, Santi S, Merigliano S, Sileni VC, Koussis H, Zaninotto G, Bonavina L, Peracchia A (2001) Only pathologic complete response to neoadjuvant chemotherapy improves significantly the long term survival of patients with resectable esophageal squamous cell carcinoma: final report of a randomized, controlled trial of preoperative chemotherapy versus surgery alone. Cancer 91: 2165–2174 11391598

[bib2] Bakkenist CJ, Kastan MB (2003) DNA damage activates ATM through intermolecular autophosphorylation and dimmer dissociation. Nature 421: 499–506 12556884 10.1038/nature01368

[bib3] Bartkova J, Horejsi Z, Koed K, Kramer A, Tort F, Zieger K, Guldberg P, Sehested M, Nesland JM, Lukas C, Orntoft T, Lukas J, Bartek J (2005) DNA damage response as a candidate anti-cancer barrier in early human tumorigenesis. Nature 434: 864–870 15829956 10.1038/nature03482

[bib4] Becker K, Mueller JD, Schulmacher C, Ott K, Fink U, Busch R, Bottcher K, Siewert JR, Hofler H (2003) Histomorphology and regression grading in gastric carcinoma treated with neoadjuvant chemotherapy. Cancer 98: 1521–1530 14508841 10.1002/cncr.11660

[bib5] Brücher BLDM, Becker K, Lordick F, Fink U, Sarbia M, Stein H, Busch R, Zimmermann F, Molls M, Hofler H, Siewert JR (2006) The clinical impact of histopathologic response assessment by residual tumor cell quantification in esophageal squamous cell carcinomas. Cancer 106: 2119–2127 16607651 10.1002/cncr.21850

[bib6] Brücher BLDM, Sarbia M, Oestreicher E, Molls M, Burian M, Biemer E, Stein HJ (2007) Squamous cell carcinoma and Zenker diverticulum. Dis Esoph 20: 75–78 10.1111/j.1442-2050.2007.00648.x 17227315

[bib7] Brücher BLDM, Stein HJ, Zimmermann F, Werner M, Sarbia M, Busch R, Dittler HJ, Molls M, Fink U, Siewert JR (2004) Responders benefit from neoadjuvant RTx/CTx in esophageal squamous cell carcinoma: results of a prospective phase-II trial. Eur J Surg Oncol 30: 963–971 15498642 10.1016/j.ejso.2004.06.008

[bib8] Enzinger PC, Mayer RJ (2003) Esophageal cancer. N Engl J Med 349: 2241–2252 14657432 10.1056/NEJMra035010

[bib9] Gao C, Nakajima T, Taya Y, Tsuchida N (1999) Activation of p53 in MDM2-overexpressing cells through phosphorylation. Biochem Biophys Res Commun 264: 860–864 10544021 10.1006/bbrc.1999.1611

[bib10] Gibson MK, Abraham SC, Wu TT, Burtness B, Heitmiller RF, Heath E, Forastiere A (2003) Epidermal growth factor receptor, p53 mutation, and pathological response predict survival in patients with locally advanced esophageal cancer treated with preoperative chemoradiotherapy. Clin Cancer Res 9: 6461–6468 14695149

[bib11] Hickey K, Grehan D, Reid IM, O'Briain S, Walsh TN, Hennessy TP (1994) Expression of epidermal growth factor receptor and proliferating cell nuclear antigen predicts response of esophageal squamous cell carcinoma to chemoradiotherapy. Cancer 74: 1693–1698 7915963 10.1002/1097-0142(19940915)74:6<1693::aid-cncr2820740609>3.0.co;2-#

[bib12] Ilson DH (2004) New developments in the treatment of esophageal cancer. Clin Adv Hematol Oncol 2: 97–104 16163169

[bib13] Ito T, Kaneko K, Makino R, Ito H, Konishi K, Kurahashi T, Kitahara T, Mitamura K (2001) Prognostic value of p53 mutations in patients with locally advanced esophageal carcinoma treated with definitive chemoradiotherapy. J Gastroenterol 36: 303–311 11388392 10.1007/s005350170095

[bib14] Kastan MB, Bartek J (2004) Cell-cycle checkpoints and cancer. Nature 432: 316–323 15549093 10.1038/nature03097

[bib15] Krasna MJ, Mao YS, Sonett JR, Tamura G, Jones R, Suntharalingam M, Meltzer SJ (1999) P53 gene protein overexpression predicts results of trimodality therapy in esophageal cancer patients. Ann Thorac Surg 68: 2021–2024, discussion 2024–2025 10616970 10.1016/s0003-4975(99)01146-7

[bib16] Lerut T, Coosemans W, Leyn PD, Van Raemdonck D, Deneffe G, Decker G (1999) Treatment of esophageal carcinoma. Chest 116: 463–465 10.1378/chest.116.suppl_3.463s 10619509

[bib17] Miller AB, Hoogstraten B, Staquet M, Winkler A (1981) Reporting results of cancer treatment. Cancer 47: 207–214 7459811 10.1002/1097-0142(19810101)47:1<207::aid-cncr2820470134>3.0.co;2-6

[bib18] Miyazono F, Metzger R, Warnecke-Eberz U, Baldus SE, Brabender J, Bollschweiler E, Doerfler W, Mueller RP, Dienes HP, Aikou T, Hoelscher AH, Schneider PM (2004) Quantitative c-erbB-2 but not c-erbB-1 mRNA expression is a promising marker to predict minor histopathologic response to neoadjuvant radiochemotherapy in oesophageal cancer. Br J Cancer 91: 666–672 15213712 10.1038/sj.bjc.6601976 PMC2364782

[bib19] Pühringer-Oppermann F, Stahl M, Keller G, Sarbia M (2006) Lack of prognostic impact of p53 gene mutation and p53 phosphorylation at serine 15 in multimodally treated adenocarcinomas of the gastroesophageal junction. J Cancer Res Clin Oncol 132: 433–438 16538517 10.1007/s00432-006-0085-9 PMC12161083

[bib20] Ribeiro UJR, Finkelstein SD, Safatle-Ribeiro AV, Landreneau RJ, Clarke MR, Bakker A, Swalsky PA, Gooding WE, Posner MC (1998) P53 sequence analysis predicts treatment response and outcome of patients with esophageal carcinoma. Cancer 83: 7–18 9655287

[bib21] Sarbia M, Stahl M, Fink U, Willers R, Seeber S, Gabbert HE (1998) Expression of apoptosis-regulating proteins and outcome of esophageal cancer patients treated by combined therapy modalities. Clin Cancer Res 12: 2991–2997 9865911

[bib22] Seitz JF, Perrier H, Monges G, Giovannini M, Gouvernet J (1995) [Multivariate analysis of the prognostic and predictive factors of response to concomitant radiochemotherapy in epidermoid cancers of the esophagus. Value of immunodetection of protein p53; in French. Gastroenterol Clin Biol 19: 465–474 7589997

[bib23] Shieh SY, Ikeda M, Taya Y, Prives C (1997) DNA damage-induced phosphorylation of p53 alleviates inhibition by MDM2. Cell 91: 325–334 9363941 10.1016/s0092-8674(00)80416-x

[bib24] Shimada H, Hoshino T, Okazumi S, Matsubara H, Funami Y, Nabeya Y, Hayashi H, Takeda A, Shiratori T, Uno T, Ito H, Ochiai T (2002) Expression of angiogenic factors predicts response to chemoradiotherapy and prognosis of oesophageal squamous cell carcinoma. Br J Cancer 86: 552–557 11870536 10.1038/sj.bjc.6600129 PMC2375274

[bib25] Siewert JR, Stein HJ, Feith M, Brücher BLDM, Bartels H, Fink U (2001) Histologic tumor type is an independent prognostic parameter in esophageal cancer: lessons from more than 1000 consecutive resections at a single center in the Western world. Ann Surg 234: 360–367 11524589 10.1097/00000658-200109000-00010 PMC1422027

[bib26] Soontrapornchai P, Elsaleh H, Joseph D, Hamdorf JM, House AK, Iacopetta B (1999) TP53 gene mutation status in pretreatment biopsies of oesophageal adenocarcinoma has no prognostic value. Eur J Cancer 35: 1683–1687 10674013 10.1016/s0959-8049(99)00172-0

[bib27] Wittekind C, Meyer HJ, Bootz F (2002) TNM Classification of Malignant Tumours, 6th edn, Berlin: Springer

